# Cannabis Use and Anxiety: Is Stress the Missing Piece of the Puzzle?

**DOI:** 10.3389/fpsyt.2014.00168

**Published:** 2014-11-24

**Authors:** Elizabeth C. Temple, Matthew Driver, Rhonda F. Brown

**Affiliations:** ^1^Federation University Australia, Ballarat, VIC, Australia; ^2^University of New England, Armidale, NSW, Australia; ^3^The Australian National University, Canberra, ACT, Australia

**Keywords:** cannabis, anxiety, stress, self-medication, path analysis

## Abstract

**Objective:** Comorbidity between anxiety and cannabis use is common yet the nature of the association between these conditions is not clear. Four theories were assessed, and a fifth hypothesis tested to determine if the misattribution of stress symptomology plays a role in the association between state-anxiety and cannabis.

**Methods:** Three-hundred-sixteen participants ranging in age from 18 to 71 years completed a short online questionnaire asking about their history of cannabis use and symptoms of stress and anxiety.

**Results:** Past and current cannabis users reported higher incidence of lifetime anxiety than participants who had never used cannabis; however, these groups did not differ in state-anxiety, stress, or age of onset of anxiety. State-anxiety and stress were not associated with frequency of cannabis use, but reported use to self-medicate for anxiety was positively associated with all three. Path analyses indicated two different associations between anxiety and cannabis use, pre-existing and high state-anxiety was associated with (i) higher average levels of intoxication and, in turn, acute anxiety responses to cannabis use; (ii) frequency of cannabis use via the mediating effects of stress and self-medication.

**Conclusion:** None of the theories was fully supported by the findings. However, as cannabis users reporting self-medication for anxiety were found to be self-medicating stress symptomology, there was some support for the stress-misattribution hypothesis. With reported self-medication for anxiety being the strongest predictor of frequency of use, it is suggested that researchers, clinicians, and cannabis users pay greater attention to the overlap between stress and anxiety symptomology and the possible misinterpretation of these related but distinct conditions.

Globally, cannabis is the most commonly used illicit drug (1) and anxiety is the most prevalent mental disorder (2). Large cross-sectional population-based surveys, such as the Australian *National Survey of Mental Health and Wellbeing*, the United States *National Comorbidity Survey*, and *National Epidemiological Survey of Alcohol and Related Conditions*, consistently find that cannabis users report a higher incidence of anxiety disorders and symptoms than non-cannabis users [e.g., Ref. (3–5)]. Australian data, for example, indicates a 40.5% prevalence rate for anxiety disorders in current cannabis users with a 12-month cannabis use disorders (CUD) and a rate of 20.8% for current users without CUD, in comparison to 11.2% for non-users (5). As such, the existence of comorbidity between anxiety and cannabis use is well known.

Similarly, well known is that the subjective effects of acute cannabis intoxication, which typically include feelings of euphoria and relaxation, also include feelings of anxiety and/or paranoia for a significant proportion of users (6, 7). Thus, many people report using cannabis to relieve symptoms of stress and/or anxiety, while concurrently some users report experiencing acute anxiety symptoms when intoxicated (8). Inconsistent with both of these types of user experiences, Tournier et al. (9) found no evidence for the anxiolytic (i.e., anxiety-reducing) or anxiogenic (i.e., anxiety inducing or increasing) effects of cannabis use in daily life when using an experience sampling method. Tournier et al. (9) also failed to find a significant association between state-anxiety and cannabis use, but found that use was associated with agoraphobia. Cannabis use has also been found to be related to panic disorder [e.g., Ref. (10, 11)], social phobia [e.g., Ref. (10)], and posttraumatic stress disorder [e.g., Ref. (12)], yet other studies have reported that cannabis use disorder is unrelated to anxiety disorders other than social anxiety disorder [e.g., Ref. (13)], or that associations between cannabis use and anxiety are non-significant after controlling for confounders [e.g., Ref. (14)].

These apparent contradictions suggest that there is a need to more clearly distinguish between the different types of anxiety (i.e., state-anxiety, drug-induced anxiety) that occur in the context of cannabis use. There is also clearly a need to clarify *how* cannabis use is related to the development and perpetuation of anxiety and/or how anxiety contributes to the use of cannabis. Furthermore, it is important to investigate whether the common tendency for lay people to use the words “anxiety” and “stress” interchangeably, which stems from a lack of awareness of the distinguishing features of each (15) has contributed to the mixed results evident in the literature investigating cannabis use and anxiety.

## The Relationship between Cannabis and Anxiety

Four main theories have been proposed to explain the relationship between cannabis use and anxiety (16), each of which has supporting evidence. The *common factor theory* proposes that the associations found between cannabis and anxiety exist because both have common antecedents, which may include biological, social, and environmental factors such as childhood trauma, personality, and socioeconomic adversity (16–18). This theory is supported, for example, by the findings from two longitudinal studies, the *Netherlands Mental Health Survey and Incidence Study* (19) and the *Christchurch Health and Development Study* [e.g., Ref. (20)], where associations between cannabis use and anxiety were non-significant after potential confounding factors were taken into account.

The *self-medication hypothesis* proposes the association exists because individuals experiencing anxiety are motivated to use cannabis to alleviate their negative affective symptoms (16, 18, 21). This theory is concordant with prior study results indicating that stress relief, relaxation, and anxiety/tension reduction are the most common reasons for cannabis use (22, 23), and that cannabis can induce anxiolytic effects (24). Further supporting evidence comes from past findings that a large proportion of individuals with comorbid anxiety and CUD experience the onset of their anxiety disorder prior to the onset of cannabis use (3). Moreover, Buckner and Carroll’s (25) finding that reductions in anxiety symptomology within a cannabis-dependent sample led to reduced cannabis use, but that reductions in cannabis use did not lead to decreased levels of anxiety, also support the self-medication hypothesis.

The third theory posits a direct *causal association* between cannabis use and anxiety, whereby the use of cannabis increases the risk of the subsequent development of an anxiety disorder (16, 18). This hypothesis is consistent with clinical observations that panic symptoms can occur during or immediately after cannabis use (26, 27), suggesting that the drug might also directly contribute to, or at least augment, anxiety symptoms (18, 28). It is also supported by findings from longitudinal studies, such as the *Victorian Adolescent Health Cohort Study* [e.g., Ref. (17, 18)], where frequent cannabis use during adolescence has been found to be associated with greater risk for subsequent anxiety disorders during adolescence and early adulthood, even after potential confounding factors were controlled statistically.

The final, somewhat unifying, theory suggests that the associations between cannabis use and anxiety can be explained by a *reciprocal feedback loop*, with simultaneous causation between cannabis use and anxiety arising from *common factors*, and where each condition leads to the exacerbation of the other through *direct causality* and/or *self-medication* (16). This theory is supported in part by the findings from Van Dam et al.’s (29) investigation of differences between clinically anxious and non-anxious heavy cannabis users (daily/near daily use for 12 months or longer). Anxious users consumed more cannabis (in grams) per week, reported more cannabis use-related problems, and had higher levels of depression and schizotypal symptomology than non-anxious users, yet the groups were matched demographically and did not differ in relation to age at onset of cannabis use, average high, or duration of use. The authors noted that these findings suggest anxiety that may be causally related to the development of abuse/dependence for heavy users of cannabis. This proposition is somewhat supported by findings from the longitudinal *CanDep* study, which followed frequent cannabis users (used >3 times per week for at least 12 months) over 3 years (10, 30).

Cross-sectional analysis of the baseline *CanDep* data comparing non-users to dependent and non-dependent users found that dependent users were more likely to experience anxiety disorders than non-dependent users and non-users, with these two latter groups reporting comparable levels of anxiety (10). Similar to Van Dam et al. (29), van der Pol et al. (10) found that the two cannabis user groups did not differ in relation to key cannabis use factors, including age of first use and onset of regular use, duration, and frequency of use. However, in contrast to Van Dam et al. (29), van der Pol et al. (10) found that dependent and non-dependent users also did not differ in relation to the quantity of cannabis used (number of joints per day, dose). Nevertheless, dependent users were more likely than non-dependent users to use cannabis alone, use for coping and expansion motives, to be experiencing a mood disorder, and to report other current substance use (10).

As such, these two cross-sectional studies (10, 29) suggest that there is a subgroup of frequent cannabis users that is more prone to experience cannabis dependence and anxiety (as well as other psychopathology) than other users with similar exposure to cannabis use. These studies do not tend to shed light on the direction and/or existence of any causal relationships between cannabis use and anxiety. This issue is, however, addressed by longitudinal findings from the *CanDep* study (30), where non-dependent frequent users were followed from baseline for 3 years to investigate the development of cannabis dependence. In this study, anxiety was not found to be predictive of dependence, nor was dependence predicted by cannabis exposure (e.g., age at onset, frequency, quantity, dose, etc.) or any of the many stable factors that are commonly considered to be risk factors for dependence (e.g., childhood adversity, demographics, etc.) that were assessed in the study. Rather, cannabis dependence was predicted by living alone, coping motives for use, and stress (measured as number of negative recent life events).

## Stress, Anxiety and Cannabis Use

Other than van der Pol et al.’s (30) study, no prior studies appear to have investigated the distinction between stress and anxiety in cannabis users. Anxiety and stress are overlapping but quite distinct states. For example, the *stress* subscale of the Depression Anxiety Stress Scales (DASS) asks respondents about tension, persistent arousal symptoms, irritability, and difficulty relaxing, whereas the *anxiety* subscale asks about symptoms of arousal/tension and *fear*-*related* symptoms and cognitions (31). Hence, while autonomic arousal is a core feature of both states, suggesting that there may be a natural continuity or overlap between the two syndromes, there are salient differences between the disorders, such that fear cognitions occur in anxiety but not in high stress conditions. In the past, researchers have experienced substantial difficulties in separating the two constructs (31), so it is likely that cannabis users may also not appreciate the salient differences between the two states.

Accordingly, we advance a fifth explanation for the associations seen between anxiety and cannabis use, the *stress-misattribution hypothesis*, which suggests that some proportion of the associations evident between anxiety and cannabis are due to users misattributing their stress symptomology, believing that they are actually symptoms of anxiety. This hypothesis fits within the self-medication hypothesis, such that users reporting self-medication to relieve anxiety symptomology are expected to in fact be self-medicating stress/tension rather than (or in addition to) anxiety symptoms. Additionally, it is posited that this hypothesis is in keeping with the reciprocal feedback loop hypothesis, with stress playing a central role, along with anxiety, in the escalation of cannabis use and, in turn, also being exacerbated by increased cannabis use.

Consistent with this hypothesis, there is evidence in the literature to suggest that cannabis users are exposed to more stressors than non-users, and dependent users to more again than non-dependent users. In a review of the literature investigating stress as a risk factor for cannabis use/misuse, Hyman and Sinha (32) identified family dysfunction, social disadvantage, and maltreatment (i.e., physical, emotional and sexual abuse, and neglect) during childhood as stressful conditions commonly found to be associated with both early onset of cannabis use and later dependence, while trauma occurring during adulthood (e.g., interpersonal violence, combat trauma) and chronic stress were similarly implicated in the development of cannabis dependence. These types of traumatic life/events and life stressors are typically reported more often by cannabis users than non-users, and by dependent users than non-dependent users [e.g., Ref. (10, 20, 33)]. The model put forward by Hyman and Sinha (32) links these stress-inducing life events/circumstances to altered stress responses and coping deficits/disruptions and then the consumption of cannabis for coping motives and an associated increased frequency of cannabis use. These changes are posited to cause neuroadaptations in the stress and reward circuits (e.g., via cannabis-related activation of the hypothalamic-pituitary-adrenal [HPA] axis and increased dopamine release), with an exacerbating cycle then eventuating, whereby chronic cannabis use is associated with maladaptive coping and poor life decisions, which lead to increased stressors/stress/distress and, thus, increased cannabis use for coping/relief. As such, this cycle of exacerbation is consistent with the reciprocal feedback loop theory.

It is possible that maladaptive coping and/or poor decision-making in everyday life may be associated with a range of other differences commonly seen between cannabis users and non-users. For example, cannabis users are more likely than non-users to be unemployed/welfare dependent and single/living alone, and more likely to report lower levels of education, income, and life and relationship satisfaction [e.g., Ref. (10, 14, 30, 34)], with these life circumstances often associated with, or indicative of, higher levels of stress/stressors in everyday life. Further to this, the diagnostic distinctions drawn between cannabis use and cannabis abuse/dependence (DSM-IV) or cannabis use disorder (DSM-5) reflect, at least in part, an escalation of stressors in an individual’s life. For example, an individual experiencing use-related social/interpersonal problems (i.e., interpersonal stress) as well as physical or psychological use-related problems (e.g., health-related stressors) meets DSM-5 criteria for cannabis use disorder (35). Thus, by definition alone, dependent users may be experiencing higher levels of stress in their everyday lives than non-dependent users.

Furthermore, the rapidly growing body of research investigating the endocannabinoid system and, specifically, its inhibitory role in the modulation of neuronal and behavioral stress responses [e.g., Ref. (36–38)], also provides support for the existence of stress-related differences between cannabis users and non-users. Essentially, the psychoactive effects resulting from cannabis ingestion are caused by the binding of exogenous cannabinoids (e.g., THC, CBD) to cannabinoid receptors (CB_1_, CB_2_) within the endocannabinoid system. In part, this impedes the ability of endogenous cannabinoids (i.e., 2-AG, AEA) to bind with these receptors, disrupting the usual functioning of the endocannabinoid system. The endocannabinoid system is central to the regulation of emotion and acute and chronic stress responses, also acting to constrain basal activation of the HPA axis (39). Consistent with this, cannabis ingestion has been found to activate the HPA axis, particularly when used in high doses, with recent research suggesting that frequent cannabis use may result in persistent hyperactivity of the HPA axis (38). Thus, increased endocannabinoid signaling is associated with reductions in stress and anxiety symptomology and, conversely, disruption of signaling is associated with stress, anxiety, and depression (40).

To summarize, epidemiological data indicate that cannabis users report greater exposure to historical stressors (e.g., childhood maltreatment) and stressful circumstances in everyday life (e.g., unemployment/welfare dependence) than never/non-users, and dependent users report higher levels of these stressors as well as cannabis use-related stressors than non-dependent users. Frequent cannabis use may alter the functioning of the endocannabinoid system, affecting the modulation of HPA axis stress responses, and, thereby, increasing cannabis users’ vulnerability to stress and anxiety. Further, it is possible that cannabis users who are experiencing stress but not anxiety, or stress and anxiety, may misattribute at least some of their stress symptoms to anxiety. As such, individuals reporting the use of cannabis for the self-medication of anxiety symptoms may actually be (at least in part) medicating symptoms of stress/tension rather than symptoms of anxiety. This would be consistent with findings that more cannabis users report using the drug to reduce stress/tension than to reduce their anxiety (22, 23).

## The Current Study

Each of the four theories outlined above should predict a different pattern of results in relation to the association between anxiety and cannabis use. The *common factors* theory suggests that cannabis use is inconsequential to the development of anxiety, whereas the *self-medication*, *direct causation*, and *reciprocal feedback loop* theories suggest differing roles for cannabis use in the development and management of anxiety symptoms. The proposed *stress-misattribution* hypothesis suggests that misidentification of stress symptomology may account for at least part of the association commonly reported between cannabis use and anxiety. Thus, in this study, we examined the relationship between stress, anxiety, and cannabis use to test the ability of these different theories to explain the commonly reported association between cannabis use and anxiety.

**Figure 1:**
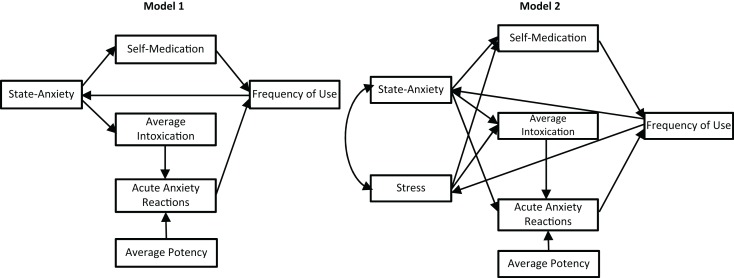
**Proposed models with reciprocal feedback loop between anxiety and cannabis use variables: (1) without stress; (2) including stress**.

Accordingly, it was hypothesized that:

1) if cannabis use and anxiety are associated solely because of *common underlying factors*:
  a) current and past users will be more likely to report lifetime anxiety than participants who have never used cannabis, with prevalence for current and past users being similar and
  b) current and past users will not differ in relation to state-anxiety, but will report higher levels than participants who have never used cannabis,
2) if the anxiety experienced by cannabis users is *caused by their cannabis use:*
  a) current users will have higher levels of state-anxiety than both past users and participants who have never used cannabis and
  b) an exposure/dose–response relationship will be evident, with levels of state-anxiety reported by current users predicted by acute anxiety reactions and/or cannabis use factors (i.e., frequency, potency, intoxication), after controlling for potential confounding variables,
3) if anxious people use cannabis to *self-medicate* their symptoms of anxiety:
  a) frequency of self-reported use of cannabis for self-medication purposes by current users will be predicted by state-anxiety, after controlling for potential confounding variables and
  b) the frequency of cannabis use reported by current users will be predicted by state-anxiety and frequency of reported use for self-medication, after controlling for potential confounding variables,
4) if the association between anxiety and cannabis use involves a *reciprocal feedback loop* path analysis will indicate good fit for a model with cannabis use for self-medication of anxiety central to the reciprocal associations between anxiety-related variables (i.e., state-anxiety, acute anxiety reactions) and cannabis use variables (i.e., frequency of use, potency, intoxication), as per Model 1 in Figure 1, and
5) if the *stress-misattribution hypothesis* posited above is relevant:
  a) current cannabis users will report higher levels of stress symptomology than both past users and participants who have never used cannabis, with the latter two groups also differing,
  b) levels stress symptomology reported by current users will be more strongly predictive of both self-medication and frequency of use than state-anxiety in relation to hypotheses 3a and 3b, and
  c) the best fit path analysis model will indicate stress symptomology, which is an integral aspect of the associations between anxiety and cannabis use variables, as per Model 2 in Figure 1.

## Method

### Participants

Participants were primarily recruited via advertisements placed on online forums and message boards relevant to cannabis use (e.g., www.cannabisculture.com) or anxiety and panic disorder (e.g., www.panicsurvivor.com). In addition, some participants were recruited through the University of New England’s (Australia) Research Participation Opportunities program, which enables first-year psychology students to receive course credit for participation in a range of available studies. The single inclusion criterion was being aged 18 years or older.

The anonymous online questionnaire was completed by 321 participants (52.0% male) aged between 18 and 71 years (*M* = 32.3 years; SD = 11.92). Most respondents were employed (61.7%) or university students (25.9%), with small proportions being either unemployed (8.9%) or retired (3.5%). The vast majority of participants (86.0%, *N* = 267) reported using cannabis at least once in their life, with 53.9% (*N* = 173) using it during past 12 months. In relation to other substance use, 43% drank alcohol at least weekly, 37.8% were current tobacco smokers, and 12.1% had used another illicit drug at least once in their lifetime.

One-fifth of the current cannabis users reported using it on a daily basis (21%), while 45% used weekly, 11% monthly, and 23% used less frequently. Cannabis using participants most commonly consumed it by smoking a joint (36.6%), with fewer typically using a bong/waterpipe (16.8%), and 31.6% reporting both methods. The most common preparation type consumed was heads/buds (64.0%), with the use of leaf (10.6%) and hash/resin (3.0%) being less common; 22.4% of cannabis users reported using a range of preparation types.

### Materials

The questionnaire collected demographic data (i.e., age, gender, employment status) and contained a range of items designed to assess various aspects of cannabis use and mental health. Qualtrics Online Survey Software was used for the questionnaire.

Participants were asked if they had ever tried *cannabis* and, if so, their *age at first use*. Those who had used cannabis were also asked to report whether or not they had used cannabis in the past 12 months. These questions were used to categorize participants into three cannabis use groups: never used, past use, and current use.

Current cannabis users were asked a number of additional questions about their use of cannabis in the previous 12 months. *Frequency of use* was assessed on a 9-point scale (1 = “only once or twice” to 9 = “every day”). *Average potency* of cannabis consumed was assessed by asking participants how often (0 = “never” to 4 = “every time”) they used cannabis with six different levels of potency (1 = “very weak” to 6 = “extremely strong”). Scores could range from 4 (“very weak” * “every time”) to 24 (“extremely strong” * “every time”). Similarly, *average level of intoxication* was calculated by asking participants how often (0 = “never” to 4 = “every time”) they experienced six different levels of intoxication (1 = “not intoxicated” to 6 = “very stoned”), with scores ranging from 4 (“not intoxicated” * “every time”) to 24 (“very stoned” * “every time”). Frequency of *acute anxiety reactions* was assessed by asking current cannabis users to report how often they had felt anxious or panicky when using cannabis (1 = “never” to 5 = “every time”), while use to *self-medicate for anxiety* was assessed by asking current users how often they had used cannabis to reduce feelings of anxiety (1 = “never” to 5 = “every time”). All of these items specified that respondents answer in relation to their use/experience of cannabis in the previous 12 months.

History of anxiety was assessed by asking respondents if they had ever experienced anxiety (1 = “never” to 6 = “this is always an issue for me”), with this item used to classify participants in relation to *lifetime anxiety* (0 = “no,” 1 = “yes”). All affected participants were asked to report the age at which they had first experienced anxiety. This item, in conjunction with reported age at first use of cannabis, was used to determine *pre-existing anxiety* (0 = “no,” 1 = “yes”). Participants were also asked to report whether there was a history of anxiety and/or depression within their family (0 = “no,” 1 = “yes”).

*State-anxiety* and *stress* were assessed using the 42-item version of the DASS (31). Participants rated the extent to which each state had been experienced over the past week using a 4-point scale (0 = “did not apply to me at all” to 3 = “applied to me very much or most of the time”). Each of the three subscales, depression, anxiety, and stress, consists of 14 items and has a maximum scoring range of 0–42, with higher scores indicating higher levels of the relevant state. Convergent and discriminant validity of the DASS is reported to be adequate and internal consistency reliabilities for the DASS subscales are high, with subscale Cronbach’s alphas ranging from 0.90 to 0.95 (41). In this study, Cronbach’s alphas for the subscales were high, ranging from 0.93 to 0.97. For the purposes of this study, only results relating to the anxiety and stress scales are reported.

### Procedure

To gain access to the online questionnaire, participants clicked on the link that was provided to them in the recruitment message. They were first presented with an information page outlining the purpose of the study and providing relevant information to enable informed consent to participate to the study. If consent was indicated, participants were then directed to the first page of the questionnaire. It took approximately 20 min for questionnaire completion. This study was conducted with full human research ethics committee approval.

### Statistical analyses

Due to the large number of hypotheses and overlapping analyses planned to test them, a summary is provided in Table 1.

**Table 1:** **Statistical analyses planned to test each theory and hypothesis**.

Theory and hypotheses	Planned analyses
1. Common underlying factors	
a. Lifetime anxiety: CU = PU > NU	Chi-square: cannabis group × lifetime anxiety
b. State-anxiety: CU = PU > NU	ANOVA: IV = cannabis group, DV = state-anxiety
2. Anxiety caused by cannabis use	
a. State-anxiety CU > PU and NU	ANOVA: IV = cannabis group, DV = state-anxiety
b. State-anxiety: exposure/dose-response for CU	Regression: IV = cannabis use factors, DV = state-anxiety
3. Self-medication	
a. Self-medication predicted by state-anxiety	Regression: IV = state-anxiety, DV = self-medication
b. Frequency of use predicted by state-anxiety and self-medication	Regression: IV = state-anxiety, self-medication DV = frequency
4. Reciprocal feedback loop	
Cannabis use for self-medication of state-anxiety central to reciprocal associations	Path analysis: Model 1
5. Stress misattribution	
a. Stress: CU > PU > NU	ANOVA: IV = cannabis group, DV = stress
b. Stress stronger predictor of self-medication and frequency of use than state-anxiety	Regression: IV = state-anxiety, stress, DV = self-medication
c. Cannabis use for self-medication of stress central to reciprocal associations	Path analysis: Model 2

*CU, current users; PU, past users; NU, never used; IV, independent variable; DV, dependent variable*.

A chi-squared analysis was used to test hypothesis 1a, with the three cannabis use groups (never, past, current) compared in relation to prevalence of lifetime anxiety (yes, no). Hypotheses 1b, 2a, and 5a were tested with two one-way analysis of variances (ANOVAs) examining cannabis use group (never, past, current) differences in state-anxiety and stress. Pearson’s bivariate correlation coefficients were calculated on data from current cannabis users to identify variables (independent and potential confounds) for inclusion in the three hierarchical multiple regression analyses that were completed to test hypotheses 2b (dependent variable [DV]: state-anxiety), 3a/5b (DV: self-medication), and 3b/5b (DV: frequency of use). A fourth hierarchical multiple regression analysis, with acute anxiety reactions as the dependent variable, was completed to assist in the development of a path analysis model. All these statistical analyses were completed using IBM SPSS Statistics 22 Software.

Finally, path analyses were completed to test hypotheses 4 and 5c. Three models were tested: state-anxiety only (Model 1, depicted in Figure 1), state-anxiety and stress (Model 2, depicted in Figure 1), and a third model that was informed by the results from the correlation and hierarchical regression analyses (Model 3, depicted in Figure 4). Path analyses were performed in IBM SPSS Amos 22 using the maximum likelihood method of estimation. In accordance with Hu and Bentler (42), good model fit was assessed using the combination of chi-squared (χ^2^ > 0.05), the Tucker–Lewis index (TLI > 0.95), comparative fit index (CFI > 0.95), and root mean square error of approximation (RMSEA < 0.05).

## Results

### Differences between cannabis use groups

As can be seen in Table 2, participants in the three cannabis use groups were found to differ in relation to gender and age, with current users younger on average than past users and the current use group containing disproportionately more males than the other two groups. Lifetime anxiety rates were also found to differ between the three user groups. Specifically, 40.0% (*n* = 18) of the participants who had never used cannabis reported having experienced anxiety at some time during their lifetime, which was significantly lower than the 67.6% (*n* = 117) of current users and 71.8% (*n* = 73) of past users who reported lifetime anxiety: χ^2^(2, *N* = 321) = 15.03, *p* = 0.001. However, no cannabis use group differences were found in relation to state-anxiety [*F*(2,290) = 0.06, *p* = 0.944, η^2^ < 0.001] or stress symptomology: *F*(2,290) = 1.71, *p* = 0.182, η^2^ = 0.012 (see Table 2).

**Table 2:** **Means and standard deviations (SD) and cannabis use group differences**.

	Never used (NU; *N* = 45)	Past use (PU; *N* = 102)	Current use (CU; *N* = 173)	All (*N* = 320)	Group differences
Gender (% male)^a^	33%	40%	64%	52%	CU > PU and NU***
Current age^b^	32.3 (13.95)	37.1 (10.71)	29.5 (11.19)	32.3 (11.92)	CU < PU***
Lifetime anxiety^a^	40%	72%	68%	65%	NU < CU and PU**
State-anxiety^b^	5.7 (9.75)	5.3 (7.26)	5.3 (6.77)	5.3 (7.39)	Nil
Stress^b^	8.6 (10.05)	10.8 (9.19)	8.7 (8.99)	9.4 (9.23)	Nil
Depression^b^	8.3 (12.06)	8.4 (10.25)	7.5 (9.56)	7.9 (10.15)	Nil
Age at anxiety onset^b^	20.1 (12.88)	19.6 (8.60)	17.4 (7.62)	18.5 (8.75	Nil
Age cannabis onset^b^	–	16.9 (3.95)	16.3 (5.07)	16.5 (4.69)	Nil
Family history anxiety^a^	50%	66%	53%	57%	Nil
Family history depression^a^	59%	75%	65%	68%	Nil

^a^Chi-squared analyses;

^b^ANOVAs; *p < 0.05, **p < 0.01, ***p < 0.001.

### Associations between variables

The inclusion of independent (IV) and potentially confounding (CV) variables in the four hierarchical multiple regressions was guided by the Pearson’s bivariate correlation analyses results for current cannabis users (see Table 3), with CVs entered at step 1 and IVs entered in subsequent steps in the analyses.

**Table 3:** **Correlations between key variables for current cannabis users**.

	1	2	3	4	5	6	7	8	9	10
State-anxiety	–									
Stress	0.690***	–								
Lifetime anxiety	0.355***	0.404***	–							
Pre-existing anxiety	0.181*	0.116	0.252**	–						
Acute anxiety reactions	0.355***	0.329***	0.257**	0.061	–					
Self-medication	0.257**	0.309***	0.398***	0.182*	−0.082	–				
Frequency of use	0.014	0.074	0.135	0.109	−0.179*	0.459***	–			
Average intoxication	0.286***	0.352***	0.131	0.076	0.355***	−0.022	−0.166*	–		
Average potency	−0.073	0.076	−0.038	−0.027	−0.031	0.106	0.262**	0.134	–	
Age at 1^st^ use	−0.006	−0.019	<0.001	0.223**	−0.151	0.072	−0.021	−0.078	−0.136	–
Current age	−0.231**	−0.095	−0.127	−0.120	−0.245**	0.033	0.099	−0.320***	0.060	0.276***
Gender	−0.034	0.133	−0.024	0.011	0.102	−0.123	−0.144	−0.024	−0.085	−0.091

*p < 0.05; **p < 0.01; ***p < 0.001.

In the first regression analysis, a significant proportion of variance in state-anxiety was predicted by current age, pre-existing anxiety, average intoxication, and acute anxiety reactions: *R* = 0.43, Adj. *R*^2^ = 0.16, *F*(4,126) = 7.02, *p* < 0.001. However, acute anxiety reactions (β = 0.27, *p* = 0.003) were the only significant predictors in the final model (see Table 4). In the second regression analysis, the use of cannabis for self-medication of anxiety was significantly predicted by pre-existing anxiety, state-anxiety, and stress: *R* = 0.35, Adj. *R*^2^ = 0.10, *F*(3,150) = 6.79, *p* < 0.001. While state-anxiety was a significant predictor in the second model of this regression (β = 0.23, *p* = 0.004), it was no longer significant (β = 0.06, *p* = 0.587) once stress was entered. As such, stress (β = 0.25, *p* = 0.021) was the only significant predictor of self-medication in the final model (see Table 4).

**Table 4:** **Summary of hierarchical regression analyses for variables predicting state-anxiety, self-medication, frequency of use, and acute anxiety reactions**.

	Model 1			Model 2			Model 3		
	*B*	*SE B*	β	*B*	*SE B*	β	*B*	*SE B*	β
State-anxiety	*R*^2^ = 0.080; *F* for Δ*R*^2^ = 5.36**			*R*^2^ = 0.125; *F* for Δ*R*^2^ = 6.45*			*R*^2^ = 0.187; *F* for Δ*R*^2^ = 9.24**		
Current age	−0.13	0.05	−0.21*	−0.09	0.05	−0.15	−0.07	0.05	−0.11
Pre-existing anxiety	2.32	1.22	0.17	2.07	1.19	0.15	2.14	1.16	0.15
Average intoxication				0.65	0.26	0.23*	0.41	0.26	0.14
Acute anxiety reactions							1.78	0.59	0.27**
Self-medication	*R*^2^ = 0.038; *F* for Δ*R*^2^ = 5.85*			*R*^2^ = 0.089; *F* for Δ*R*^2^ = 8.37**			*R*^2^ = 0.122; *F* for Δ*R*^2^ = 5.43*		
Pre-existing anxiety	0.53	0.22	0.19*	0.42	0.22	0.15	0.42	0.22	0.15
State-anxiety				0.04	0.15	0.23**	0.01	0.02	0.06
Stress							0.04	0.02	0.25*
Frequency of use	*R*^2^ = 0.029; *F* for Δ*R*^2^ = 4.05*			*R*^2^ = 0.221; *F* for Δ*R*^2^ = 33.52***					
Acute anxiety reactions	−0.47	0.23	−0.17*	−0.37	0.21	−0.13			
Self-medication				0.96	0.16	0.44***			
Acute anxiety reactions	*R*^2^ = 0.058; *F* for Δ*R*^2^ = 5.74*			*R*^2^ = 0.124; *F* for Δ*R*^2^ = 3.47*			*R*^2^ = 0.141; *F* for Δ*R*^2^ = 0.90		
Current age	−0.02	0.01	−0.24*	−0.01	0.01	−0.17	−0.01	0.01	−0.16
Frequency of use				−0.05	0.04	−0.12	−0.06	0.04	−0.15
Average intoxication				0.09	0.05	0.22*	0.07	0.05	0.16
State-anxiety							0.01	0.02	0.06
Stress							0.01	0.02	0.09

*p < 0.05; **p < 0.01; ***p < 0.001.

The third regression analysis, investigating frequency of cannabis use, contained only two variables: acute anxiety reactions and use of cannabis for self-medication of anxiety. Together these variables accounted for 21% of variance in frequency of cannabis use [*R* = 0.47, Adj. *R*^2^ = 0.21, *F*(2,138) = 19.27, *p* < 0.001]; however, only self-medication (β = 0.44, *p* < 0.001) explained a significant proportion of variance in the final model. In the final regression analyses, the five predictor variables explained 9% of variance in acute anxiety reactions to cannabis use: *R* = 0.38, Adj. *R*^2^ = 0.09, *F*(2,148) = 2.29, *p* = 0.016. Nevertheless, none of the variables independently explained a significant amount of variance in the final model (see Table 4).

### Model testing

**Figure 2:**
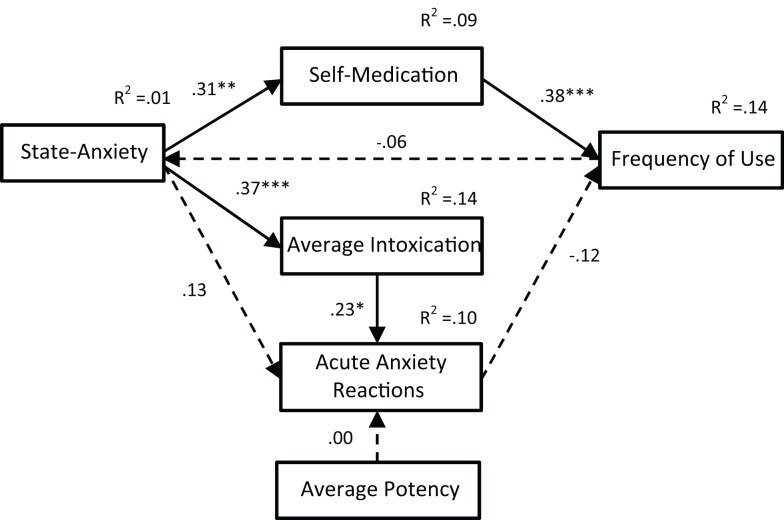
**Path analysis of Model 1**. With the exception of noted *R*^2^ values, all values are standardized regression weights (β), dashed lines indicate non-significant associations. Model fit: χ^2^(7) = 11.12, *p* = 0.133; TLI = 0.804; CFI = 0.909; RMSEA = 0.079 (0.000, 0.162). *p < 0.05, **p < 0.01, ***p < 0.001.

**Figure 3:**
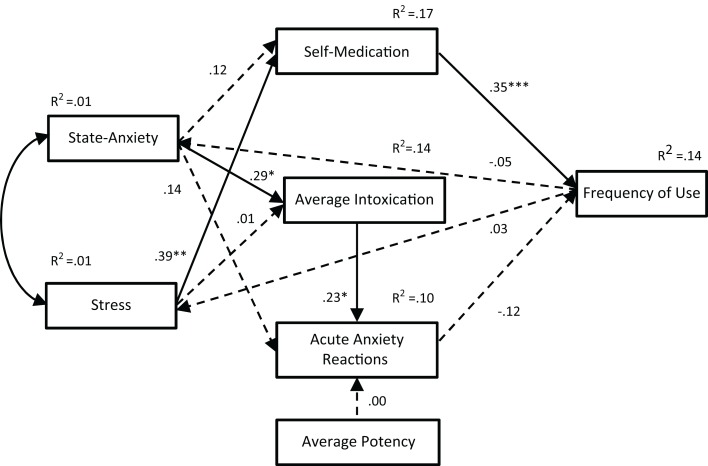
**Path analysis of Model 2**. With the exception of noted *R*^2^ values, all values are standardized regression weights (β), dashed lines indicate non-significant associations. Model fit: χ^2^(9) = 13.23, *p* = 0.152; TLI = 0.924; CFI = 0.967; RMSEA = 0.070 (0.000, 0.146). *p < 0.05, **p < 0.01, ***p < 0.001.

**Figure 4:**
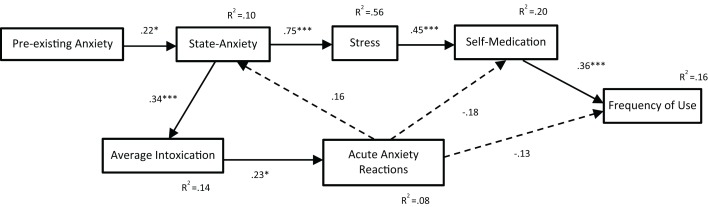
**Path analysis of Model 3**. With the exception of noted *R*^2^ values, all values are standardized regression weights (β), dashed lines indicate non-significant associations. Model fit: χ^2^(12) = 9.96, *p* = 0.620; TLI = 1.027; CFI = 1.000; RMSEA = 0.000 (0.000, 0.089). *p < 0.05, **p < 0.01, ***p < 0.001.

Path analyses were completed to test three alternate models of the relationships between anxiety and cannabis use variables. The first model included state-anxiety but not stress (see Figure 2). The second model included both state-anxiety and stress (see Figure 3). The third model also included both state-anxiety and stress, but differed from the second model in that it was informed by the correlation and hierarchical multiple regression findings. As such, pre-existing anxiety was included as a variable and average potency was excluded. Furthermore, the paths between variables in this third model were guided by suggested mediation effects indicated within the regression results. That is, when an IV was significant in one regression model and then became non-significant after the addition of a second IV, this suggested that the relationship between the first IV and the DV in question may be mediated by the second IV. For example, in the second regression analyses, state-anxiety was a significant predictor of self-medication until stress was entered, thus suggesting that the association between state-anxiety and self-medication is mediated by stress. This third model is illustrated in Figure 4.

The fit indices for all three path analysis models are displayed in Table 5. A non-significant chi-squared result indicates that the proposed model is consistent with the data; all three path models met this criterion for good model fit. The TLI typically ranges from 0 to 1 (values occasionally fall slightly outside this range) with values greater than 0.95 indicative of a good fit; only Model 3 met this criterion. Similarly, the CFI ranges from 0 to 1 with values above 0.95 being indicative of good fit; Models 2 and 3 met this criterion. RMSEA values also range from 0 to 1; however, values less than 0.05 are considered indicative of good fit; only Model 3 met this criterion. These results indicate that Models 1 and 2 do not fit adequately with the data. In contrast, the fit indices for Model 3 indicate an excellent fit.

**Table 5:** **Fit indices for the path analysis models**.

	Model 1	Model 2	Model 3
	State-anxiety	State-anxiety and stress	State-anxiety and stress with mediation
χ^2^	χ^2^(7) = 11.12, *p* = 0.133	χ^2^(9) = 13.23, *p* = 0.152	χ^2^(12) = 9.96, *p* = 0.620
TLI	0.804	0.924	1.027
CFI	0.909	0.967	1.000
RMSEA	0.079 (0.000, 0.162)	0.070 (0.000, 0.146)	0.000 (0.000, 0.089)

The path analysis results for Model 3 indicate that: (1) pre-existing anxiety is associated with increased levels of state-anxiety (β = 0.22, *p* = 0.026), which is then associated with increased levels of stress symptomology (β = 0.75, *p* < 0.001), with this in turn associated with increased frequency of use of cannabis to self-medicate for anxiety symptoms (β = 0.45, *p* < 0.001), leading to increased frequency of cannabis use (β = 0.36, *p* < 0.001); (2) state-anxiety is associated with higher average levels of intoxication (β = 0.34, *p* < 0.001), which is associated with more frequent acute anxiety reactions (β = 0.23, *p* = 0.025). While non-significant, the positive association between acute anxiety reactions and state-anxiety (β = 0.16, *p* = 0.120) and the negative associations with self-medication (β = -0.18, *p* = 0.056) and frequency of use (β = -0.13, *p* = 0.168) contributed nonetheless to the amount of variance explained within the model.

## Discussion

This study aimed to clarify the nature of the associations evident between cannabis use and anxiety variables and to explore the role of stress within these relationships. To do this, key elements of each of the four theories commonly posited to explain the relationship between cannabis use and anxiety were tested. Additionally, a fifth possible explanation for the associations evident between cannabis use and anxiety was posited, with the stress-misattribution hypothesis based on the possibility that stress/tension symptomology could be misconstrued as anxiety, thus contributing, at least in part, to the high levels of comorbidity reported between anxiety and cannabis use. A large number of hypotheses were tested, with mixed results. These will be discussed in turn, and are summarized in Table 6.

**Table 6:** **Summary of findings in relation to each theory and hypothesis**.

Theory and Hypotheses	Outcome
1. Common underlying factors
a. Lifetime anxiety: CU = PU > NU	Supported
b. State-anxiety: CU = PU > NU	Partial: CU = PU = NU
2. Direct causation
a. State-anxiety CU > PU and NU	Rejected: CU = PU = NU
b. State-anxiety: exposure/dose-response for CU	Partial: only intoxication, but became non-significant when acute anxiety reactions added as IV
3. Self-medication
a. Self-medication predicted by state-anxiety	Partial: became non-significant when stress added as IV
b. Frequency of use predicted by state-anxiety and self-medication	Partial: predicted by self-medication, not state-anxiety (or stress)
4. Reciprocal feedback loop
Cannabis use for self-medication of state-anxiety central to reciprocal associations	Rejected: Model 1 met only one of the four fit criteria
5. Stress misattribution
a. Stress: CU > PU > NU	Rejected: CU = PU = NU
b. Stress stronger predictor of self-medication and frequency of use than state-anxiety	Partial: for self-medication but not frequency of use
c. Cannabis use for self-medication of stress central to reciprocal associations	Rejected: Model 2 met two of the four fit criteria
d. Adjusted model, informed by correlation and regression findings	Supported: Model 3 met all four fit criteria

*CU, current users; PU, past users; NU, never used; IV, independent variable; DV, dependent variable*.

The *common factors theory* suggests that cannabis use and anxiety are unrelated, with apparent associations simply the by-product of underlying factor/s that lead to the development of cannabis use and anxiety independently (16–18). The veracity of this theory was tested by comparing the prevalence rates of lifetime anxiety across three cannabis use groups: never used, past use, and current use. In line with the hypothesis, the prevalence of lifetime anxiety was significantly lower in the never used group than for past and current use groups, with the latter two groups having similar levels of prevalence. This finding is consistent with reported differences in comorbidity commonly reported from large epidemiological studies for non-and current users [e.g., Ref. (3–5)]. The second hypothesis testing the common factors theory was also supported, with current and past cannabis users found to be experiencing similar levels of state-anxiety. The lack of difference between these groups suggests that current exposure to cannabis does not increase levels of state-anxiety and vice versa. Together these findings support the common factor theory by indicating that the tendency to use cannabis and experience anxiety is highly comorbid but, with state-anxiety found not to be associated with current cannabis use, it is suggested that such comorbidity is associated with a common underlying factor (e.g., childhood adversity or maltreatment).

It is important to note, however, that to be consistent with the core argument of this theory, individuals who have never used cannabis should report lower levels of state-anxiety on average than both current and past cannabis users. This was not found in the present study. Further to this, if comorbidity between lifetime anxiety and cannabis use is due to common factors occurring during childhood/adolescence that are disproportionately experienced by cannabis users (past and current) in comparison to never users, then we would also expect to see the onset of any anxiety occurring at an earlier age for individuals who had used cannabis than for individuals who had not. This was also not evident for the present sample. As such, the common factors theory is partially supported by these results, yet it seems that the assumed distal events/circumstances responsible for independently increasing the incidence of lifetime anxiety and cannabis use for some individuals may not result in ongoing repercussions that lead to subsequent increased levels of state-anxiety.

Similarly, reported stress symptomology did not differ significantly between the three cannabis use groups. These findings may suggest that the current cannabis users in the present study were no more afflicted by proximal or distal stressors than past users or those who had never used cannabis, which would be inconsistent with past research [e.g., Ref. (10, 14, 20, 30, 33, 34)]. However, as the participants were not specifically asked about life stressors (current or past), as per van der Pol et al. (30), we cannot rule out the possibility that there were unassessed group differences, such that past users and those who had never used cannabis may have been exposed to a similar or greater number stressors than the current cannabis users. Nevertheless, this finding is not consistent with the *stress-misattribution hypothesis* put forward in this paper.

The *direct causation theory* proposes that the association between cannabis use and anxiety is causal, with cannabis use causing anxiety in otherwise unaffected individuals (16, 18). For this theory to hold, we would expect to see an exposure/dose relationship between cannabis use and anxiety. Two hypotheses were proposed to test this. As noted above, the first of these, that current cannabis users would report higher levels of state-anxiety than both past and never used groups, was not upheld for the present sample. This finding suggests that current exposure to cannabis use is not associated with increased state-anxiety. Similarly, the lack of group differences discussed above in relation to stress symptomology suggests that current exposure to cannabis is not associated with increased stress/tension.

The second hypothesis involved investigating cannabis dose-related variables (i.e., frequency, potency, and intoxication) and acute anxiety reactions as predictors of state-anxiety. Interestingly, bivariate analyses indicated that neither frequency of use or average potency was significantly related to state-anxiety. Intoxication was found to account for a significant amount of variance in state-anxiety, after controlling for current age and pre-existing anxiety, in the regression analyses. However, this association was no longer significant after acute anxiety reactions was entered into the analysis, with this variable being the only significant predictor of state-anxiety in the final regression model. Given the moderately strong bivariate association indicated between level of intoxication and acute anxiety reactions, and the known links between them [e.g., Ref. (6, 43)], this result is not altogether surprising. Nevertheless, these findings suggest that there is not a direct causal relationship between cannabis use and state-anxiety but, rather, that higher levels of intoxication can induce acute anxiety reactions, which may then lead to increased levels of state-anxiety for some users – acute anxiety reactions are estimated to occur in 20–30% of users (6).

The *self-medication hypothesis* posits that the association between cannabis use and anxiety is due to anxious individuals using cannabis to relieve their anxiety symptoms (16, 18, 21). If this is the case, then state-anxiety should be positively associated with, and predictive of, the frequency with which cannabis is used specifically to relieve symptoms of anxiety. This hypothesis was partially upheld, with state-anxiety accounting for a significant proportion of variance in self-medication after controlling for pre-existing anxiety. However, once stress was entered into the regression analysis, state-anxiety was no longer significant. While this finding is evidently related to the large overlap in variance between state-anxiety and stress (*R*^2^ = 0.56), it is also suggestive of a mediation effect, whereby the effects of state-anxiety on self-medication are mediated by the effects of stress – self-medication was more strongly associated with stress (*r* = 0.31) than state-anxiety (*r* = 0.26) in the bivariate analyses.

The second hypothesis proposed to test the self-medication hypothesis, that frequency of cannabis use would be predicted by state-anxiety and use for self-medication, was also partially upheld. As noted above, state-anxiety was not associated with frequency of use; however, there was a strong positive association indicated between self-medication and frequency of use. Putting these findings together, it appears that any impact of state-anxiety (or stress) on frequency of use comes by way of self-medication. That is, individuals experiencing state-anxiety and/or stress who use cannabis to relieve their symptomology tend to use cannabis more frequently than unaffected/less affected individuals, and it is the stated use of cannabis for such self-medication purposes that seem to drive frequency of use rather than actual levels of symptomology. Hence, there is some support here for the veracity of the self-medication hypothesis, but it appears that an individual’s belief that they are using cannabis to relieve anxiety symptoms is more indicative of their frequency of use than the actual severity of the symptomology for which they are self-medicating. Additionally, they are more likely to be self-medicating symptoms of stress than of state-anxiety.

Evidently, these associations between anxiety, stress, and self-medication provide support for the *stress-misattribution hypothesis* posited in the current paper, being consistent with the idea that cannabis users may be misattributing symptoms of stress/tension to anxiety. That is, affected individuals may believe that they are using cannabis to relieve symptoms of anxiety, and thus report use for the self-medication of this disorder, while, in fact, what they are experiencing are symptoms of stress (e.g., tension, persistent arousal symptoms, irritability, and difficulty relaxing) as well as, or instead of, symptoms of anxiety [e.g., arousal/tension and fear-related symptoms and cognitions; (31)]. It should be noted, however, that stress was not found to be associated with frequency of cannabis use. Thus, even though cannabis users may misidentify the condition for which they are self-medicating, experiencing more severe stress/tension symptomology was not found to be directly associated with increased frequency of cannabis use.

Path analyses were used to test the *reciprocal feedback loop* hypothesis, which posits that cannabis use and anxiety result from common factors but then act to exacerbate each other through direct causality and/or self-medication (16). Two models were tested, one with (Model 1) and one without (Model 2) stress included as a variable. In Model 1, two significant paths were indicated: (i) from state-anxiety to average intoxication to acute anxiety reactions and (ii) from state-anxiety to self-medication to frequency of use. However, paths from state-anxiety and average potency to acute anxiety reactions and from frequency of use to state-anxiety were not significant. Furthermore, this model only met one of the four fit criteria. Model 2 was a better fit with the data, meeting two of the criteria, suggesting that the addition of stress to the model was an improvement. While the path from state-anxiety to average intoxication to acute anxiety reactions remained significant in this second model, the association between state-anxiety and self-medication in the second path was no longer significant. Rather, the significant path ran from stress to self-medication to frequency of use. Nevertheless, the majority of associations between variables proposed in this variable was non-significant, and the model was not deemed to be a good fit for the data. Therefore, a third model was developed, with the correlation and hierarchical regression results used for guidance.

In Model 3, the pathway from state-anxiety to average intoxication to acute anxiety reactions remained significant, but was lengthened with the addition of a positive association from pre-existing anxiety to state-anxiety. The second pathway was also modified, now running from pre-existing anxiety to state-anxiety to stress to self-medication to frequency of use. Pathways from acute anxiety reactions to state-anxiety (positive), self-medication (negative), and frequency of use (negative) were not found to be significant. Thus, Model 3 suggests that pre-existing anxiety (i.e., onset of anxiety prior to any cannabis use) is associated with higher levels of state-anxiety, which is then associated with higher levels of stress symptomology, leading individuals to self-medicate with cannabis and use cannabis more frequently. Additionally, it is suggested that higher levels of state-anxiety are associated with higher average levels of intoxication, which increases the frequency with which acute anxiety reactions are experienced. This model was found to be an excellent fit with the data, exceeding suggested cutoffs for all four fit indices (42).

This model provides some support the self-medication hypothesis, on the proviso that self-medication is primarily for stress symptomology, but does not support the direct causation theory. If pre-existing anxiety is considered a possible indicator of adverse events/circumstances in childhood/early adolescence, the model could be deemed to be somewhat consistent with the common factors theory. However, as this model does not include any variable that is representative of early cannabis use (age at onset of cannabis use was not significantly associated with any other cannabis variables, state-anxiety, self-medication, acute anxiety responses, or stress), the analysis cannot reasonably be considered to assess the common factors theory in any meaningful way. Nevertheless, Model 3 is somewhat consistent with the reciprocal feedback loop theory, indicating that state-anxiety, and through its pre-existing anxiety, plays a role in the escalation of cannabis use via stress and self-medication, while also playing a role in the exacerbation of acute anxiety reactions via increased average levels of intoxication. It is important to note that, in the model, neither frequency of use nor average intoxication was found to be predictive of state-anxiety, stress, or self-medication and associations between acute anxiety reactions and state-anxiety and self-medication were not significant. Hence, these findings are not in keeping with the theory’s central argument that cannabis use exacerbates state-anxiety.

The model does support the stress-misattribution hypothesis, suggesting that participants reporting self-medication of anxiety were likely to be treating stress symptomology instead of, or as well as, anxiety symptomology. Such an interpretation is consistent with prior study results indicating that the most common reason for cannabis use is to relieve stress/tension and anxiety [e.g., Ref. (8, 22)]. Further to this, Model 3 is consistent with the posited mood amplification effects of cannabis, which suggests that people with underlying anxieties may be especially vulnerable to experience acute adverse drug effects (43). The model is also concordant with study results indicating that anxiety may occur during or after cannabis intoxication (27, 44). Additionally, the results are consistent with Van Dam et al.’s (29) finding that clinically anxious heavy drug users exhibited greater drug use than non-anxious heavy drug users.

A number of limitations may, however, have lessened the veracity of the study results. First, an online survey methodology was used to capture self-reported anxiety symptoms from a general population, rather than clinical, sample, thus limiting the generalizability of the findings. Further to this, clinical anxiety diagnosis details were not collected or verified. Second, while current users were asked to report the potency of the cannabis they typically consumed, there was no opportunity for them to report the actually effects of use (beyond intoxication) to reflect known differences in effects associated with different cannabis species/breeds/hybrids or growing techniques (e.g., high vs low THC and CBD content, hydroponic vs naturally grown, etc.). Further to this, it is possible that the variations in potency encountered by cannabis users could increase the likelihood of anxiety-related experiences of use, such as when there is a higher than expected level of THC, which is not accounted for by the users (i.e., through titration of dose). Third, it is possible that some participants may have mixed tobacco with their cannabis or used other substances concurrently, potentially confounding the observed findings. Fourth, while the path models appear to suggest causal pathways between the variables, the fact that the data were cross-sectional means that causal inferences cannot be drawn. Furthermore, as there is a large range of variables that have been found in past studies to be associated in some way with cannabis use and/or anxiety that were not assessed in this study, it is possible that important factors may have been overlooked.

Nevertheless, the results of this study have important implications for the prevention and treatment of anxiety/stress disorders in cannabis users. These may also be of some benefit in relation to cannabis use-related panic attacks, which could be similarly related to stress misattribution. First, while cannabis users often report using the drug to relieve anxiety, they may actually be self-medicating symptoms of stress, the symptoms of which are readily treated by a range of well-accepted stress-reduction techniques. For example, highly stressed cannabis users could be provided with alternate stress-reduction techniques (i.e., relaxation training, physical exercise, mindfulness exercises) to reduce their symptoms of stress/anxiety and prevent the escalation in cannabis use that appears to be associated with its use for self-medication purposes. Second, cannabis users with pre-existing or current anxiety may be particularly vulnerable to experience the anxiogenic effects of cannabis, especially if they get highly intoxicated when the consume cannabis. Such individuals could be advised to restrict sessional intake/dose to reduce the likelihood of experiencing acute cannabis-related anxiety reactions. Furthermore, as two different anxiety-related paths were indicated, though both including the link from pre-existing anxiety to state-anxiety, treatments could be tailored to reflect that individuals vulnerable to experiencing acute anxiety reactions to cannabis use do not appear to be the same individuals who are using cannabis for self-medication purposes.

In summary, the findings provided some support for all of the theories, with the exception of the direct causation theory. However, none of the theories was fully supported. The common factors theory was supported by the finding that participants who had never used cannabis were less likely to report lifetime anxiety than either past or current cannabis users, but was not consistent with the finding that the three groups did not differ in relation to age at onset of anxiety, or their levels of state-anxiety or stress. The self-medication theory holds only if it is broadened to account for the treatment of stress symptoms and also acknowledges that cannabis users’ belief that they are self-medicating anxiety is a stronger predictor of frequency of use than the actual severity of the anxiety symptomology they report they are relieving. The reciprocal feedback loop theory was only partially supported by the link from state-anxiety to intoxication to acute anxiety responses. However, with frequency of use not being predictive of state-anxiety, there was no clear feedback loop to support the premise that cannabis use exacerbates state-anxiety. These results suggest that the relationship between cannabis use and anxiety is complex and likely to be obscured, at least in part, by the misidentification of overlapping symptoms of stress and anxiety. As such, the posited stress-misattribution hypothesis was partially supported.

## Conflict of Interest Statement

The authors declare that the research was conducted in the absence of any commercial or financial relationships that could be construed as a potential conflict of interest.
